# Physicochemical Properties and Effect of Processing Methods on Mineral Composition and Antinutritional Factors of Improved Chickpea* (Cicer arietinum L.)* Varieties Grown in Ethiopia

**DOI:** 10.1155/2019/9614570

**Published:** 2019-06-02

**Authors:** Ebisa Olika, Solomon Abera, Asnake Fikre

**Affiliations:** ^1^Department of Food Science and Nutrition, Wollega University, Shambu Campus, P.O. Box 38, Shambu, Ethiopia; ^2^Department of Food Science and Technology, School of Food Science, Postharvest Technology and Process Engineering, Haramaya University, P.O. Box 138, Haramaya, Ethiopia; ^3^Chickpea Regional Breeder International Crops Research Institute for the Semi-Arid Tropic, P.O. Box 5689, Addis Ababa, Ethiopia

## Abstract

Chickpea (*Cicer arietinum L*.) is an important pulse crop grown and consumed all over the world because it is a good source of carbohydrates and protein. However, presence of antinutritional components restricts its use by interfering with digestion of macronutrients* during consumption. *Therefore, the objective of this study was to evaluate* physicochemical* properties and effect of processing methods on antinutritional factors and mineral composition of improved chickpea varieties (Natoli of Desi and Arerti of Kabuli) grown in Ethiopia. The experiment was factorial with complete randomized design. The result indicated that physicochemical properties such as seed mass, seed density, hydration capacity, swelling capacity, unhydrated seeds, and cooking time of Arerti and Natoli chickpeas had 260.69 and 280.65 g/1000 seeds, 3.48 and 3.61g/ml, 1.07 and 1.03 g/g, 2.12 and 1.94ml/g, 1.64 and 14.75%, and 21.00 and 246.33 min, respectively. After processing, Zn, Fe, and Ca contents of improved chickpea varieties had 4.48 to 5.85mg/kg, 8.52 to 10.17mg/kg, and 536.56 to 1035mg/kg, respectively. The antinutritional factors, tannin and phytic acid, in the raw chickpeas were reduced to 25 to 82.25% and 5.89 to 57.35%, respectively. The results of the current study showed that Arerti of Kabuli variety showed low antinutritional factors and better physicochemical properties, specifically low cooking time, than Natoli of Desi variety. All processing methods were effective in reduction of antinutritional factors; however, boiling was found to be the best for reduction of antinutritional factors.

## 1. Introduction

Legumes are important sources of protein in the diets of millions of people in the world [1]. Among the different legumes, chickpea (*Cicer arietinum L*.) is categorized in Fabaceae (Leguminosae) family, one of the oldest and most widely consumed legumes in the world, and it is a staple food crop particularly in tropical and subtropical areas [2]. A large number of grown chickpea cultivars have various physical, hydrating, cooking, and parching characteristics [3]. According to the color of seed and geographic distribution, chickpea is grouped into two biotypes: Desi (Indian origin) and Kabuli (Mediterranean and Middle Eastern origin); while Kabuli cultivars have large seeds with white to cream colored seed coat, Desi cultivars have small and wrinkled seeds with brown, black, or green color [4]. Chickpea is a good and cheap source of protein for people in developing countries (especially in South Asia), who are largely vegetarian either by choice or because of economic reasons. In addition, chickpea was reported as important means of controlling bronchitis, cholera, and constipation and acids in chickpea seed are supposed to lower the blood cholesterol levels. Also, regular pulse consumption such as chickpeas prevents diabetes and reduces risks of heart disease [5].

Ethiopia is one of the major producers of chickpea and ranked sixth worldwide in 2016 and serves as a multipurpose crop [6]. In Ethiopia, the use of chickpea grains for human food has long history and they are used in different forms as green vegetable (green immature seed), “Kolo” (soaked and roasted), “nifro” (boiled seeds), and “wot” (sauces) made up of “shiro” (powdered seeds) or blended with cereals and/or legumes for preparing of infant and young children foods using traditional food processing techniques like soaking, germination, fermentation, boiling, and roasting. For preparation of infant and young children at certain community, bioavailability of macro- and micronutrients like protein, Fe, and Zn is critical beside sensory acceptability, cost for purchasing, and processing and preparing using local food items [7].

However, presence of antinutritional components restricts its use by interfering with digestion of carbohydrates and proteins. They also interfere with growth, reproduction, or health and reduce protein and carbohydrate utilization when consumed regularly even in normal amounts. Some of these factors include trypsin inhibitors, phytic acid, tannins, saponin, and haemagglutinin activity which can cause adverse physiological responses or diminish the availability of certain nutrients [8, 9].

Chickpea is known with having phytate and tannin which will bind with minerals like Fe, Zn, and protein, respectively. Subsequently, it will decrease the bioavailability and digestibility unless appropriate and affordable processing techniques are implemented, and such antinutritional problems can be reduced by processing techniques such as dehulling, soaking, boiling, germinating, and roasting [9, 10]. The consumption of processed chickpea provides consumers with valuable nutrition and potential health benefits [11].

The materials selected for this study were improved varieties of Natoli and Arerti with superior performance at national and regional level in terms of production and productivity and adaptation to both biotic stress (disease, insect, and weed) and abiotic stress (particularly terminal drought) and widely adapted by farmers [12]. Their physicochemical properties of raw improved chickpea seed varieties and effect of processing methods on mineral composition and antinutritional factors of improved chickpea varieties* (Natoli of Desi and Arerti of Kabuli) grown in Ethiopia *have not been analyzed yet. Therefore, the present study was designed to fill this gap. Hence, the objective of this study was to investigate physicochemical properties and the effect of selected processing methods on mineral composition and antinutritional factors of improved chickpea varieties.

## 2. Materials and Methods

### 2.1. Materials

Six kilograms of each Natoli of Desi and Arerti of Kabuli chickpeas was collected from Debrezeit Agricultural Research Centre (DZARC), Ethiopia. The analysis was done in triplicate. The seeds were cleaned manually by removing any foreign material, damaged and broken seeds, and shriveled and insect attacked seeds. The seeds were processed by direct grinding (used as control), dehulling, soaking, germinating, boiling, and dry roasting. The processed samples except the roasted one were dried in an oven at 50°C for 24 h. All the samples including the control were ground by a laboratory mill (Cyclo sample mill model no. 3010-081p) to pass through a 75 *μ*m sieve and were kept in moisture proof plastic bag placed in air tight tin container at 4°C. The seed flours of both the control and processed samples were evaluated for nutritional composition, antinutritional, and functional properties.

### 2.2. Experimental Design

Completely randomized design (CRD) with a 2×6 factorial experiment with three replications was implemented. Totally, 36 experimental units were included in the study. Two improved chickpea varieties, Natoli of Desi and Arerti of Kabuli, were tested under five traditional processing methods (dehulling, soaking, sprouting, roasting, and boiling). Raw chickpeas of the two varieties are used as control and three replications were used.

### 2.3. Processing Methods

Raw (control): Cleaned seeds of 500 g of each of the two chickpea varieties samples were directly ground by a mill [13]. Dehulling: the cleaned seeds of 500g of each of the two chickpea varieties hulls were removed manually after soaking 600 g clean seeds of the two varieties for 6 h in distilled water at room temperature. Seeds were completely covered by water using seed-to-water ratio of 1:3 (w/v) [14]. The dehulled seeds were then dried and milled to flour. Soaking: cleaned 500g each of the two improved chickpea varieties were soaked for 12 h in distilled water at room temperature. Seeds were completely covered by water using seed-to-water ratio of 1:3 (w/v) [14]. Dry roasting: Cleaned 500 g seeds each of the two chickpea varieties were roasted by hot air oven for 30 minutes at 150°C. The cooled samples were then milled into flour [15]. Germinating: Cleaned 500 g each of the two chickpea varieties were washed and cleaned with tap water. Germinating was done according to the method used by [16]. It was performed at room temperature in a dark room. Washed chickpea varieties were soaked for 12 h in distilled water using seed-to-water ratio as 1:5(w/v). Soaked seeds were spread on moist filter paper on large plastic screen. The seeds were then covered with filter paper to reduce evaporation. The screen was placed in perforated plastic container and kept in the dark at room temperature for 72 h to germinate. The seeds were splashed every 24 h with running sodium hypochlorite at concentration of 0.01% (w/v) for 10 min to avoid mold contamination. At the end of germination period, nongerminated seeds were discarded and the germinated ones were dried at 50°C for 24 h. The dry germinated seeds were milled to flour. Boiling: Cleaned 500 g seeds of the two chickpea varieties were washed under tap water, rinsed with distilled water, placed in 2 L of distilled boiling water at 96°C, and cooked for 60 min (until soft) [13]. The boiled samples were then dried and milled.

### 2.4. Physicochemical Properties of Chickpea Raw Seed

Thousand seeds' weight: cleaned samples of each of two chickpea varieties were determined according to [17] by counting 1000 seeds using an electronic seed counter and weighing. Results were expressed as the mean of triplicates determination. Seed density: cleaned seed of each of two chickpea varieties was determined according to the method of [18]; one thousand seeds were weighed and transferred into 500 ml measuring cylinder containing 250 ml of tap water. Immediately the volume of water displaced was recorded. The mass and volume were used to calculate the seed density as g/ml. Hydration, swelling capacities, and indices*: *A 20 g samples from each were soaked in 60 ml deionized water for 24 hrs at 22°C using a 100 ml measuring cylinder [16]. After soaking, the water was drained and the peas were toweled dry with absorbent paper. The hydrated peas were weighed again to determine the increase in mass. The hydration capacity was calculated as the weight of water absorbed per gram of seeds (g of water per g of seeds):

(1) Hydration  capacity=Wt  of  after  soaking−Wt  before  soakinggWt  before  soakingg

(2) Hydration  indices=Hydration  capacityg  of  one  seed.

Swelling capacity was measured by calculating the difference in volume of deionized water displaced by peas before and after soaking (ml of water per g of seeds) [16]:

(3) Swelling  capacity=Vol  after  soaking−Vol  before  soakingg  of  seeds

(4) Swelling  indices=Swelling  capacityg  of  one  seed.

Cooking time*: *This was determined according to [16] using Mattson cooking device. A chickpea sample (50 g) was soaked in deionized water (100 ml) for 24 hours at room temperature before cooking, and the soaked samples were then positioned into each of the 25 cylindrical holes found in the cooker so that the piercing up of the 82 g rod will be in contact with the surface of the chickpea seeds. The cooker was placed into a 2 l metal beaker containing 1.5 l of boiling water. The chickpea seeds were considered as “cooked” when the tip of the brass rod passed through the seed. Cooking time (time required to cook 50% of the sample) was then recorded from the point of contact between cooker and boiling water. Unhydrated seed: Unhydrated seed was determined according to [16] percentage of number of unhydrated seeds after soaking seeds overnight to the total number of seeds which initially has a mass of 100 g.

### 2.5. Mineral Contents

Ca, Fe, and Zn were determined by atomic absorption spectrophotometer [19].

### 2.6. Antinutritional Factors

Tannin was analyzed by the modified Vanillin-HCl methanol method [20] and phytic acid was determined according to [21].

### 2.7. Statistical Analysis

Data were analyzed using SAS 9.1 software. The Analysis of Variance (ANOVA) and mean separation values were determined using least significance (LSD) and Duncan multiple range test (DMRT) and significant differences were defined at p<0.05. The results were presented as mean ± standard Error. The model used for two factors (Y=*µ*+ *Ʈ*i+*β*j+(J*β*)ij+€ijk) was used.

## 3. Results and Discussion

### 3.1. Physicochemical Properties of Raw Chickpea Varieties

The general characteristics of two improved chickpea varieties are indicated in Table 1. The thousand-seed masses of Arerti (260.69 g/1000 seeds) and Natoli (280.65 g/1000 seeds) chickpea varieties were significantly (P<0.05) different from each other. The seed mass can be considered as an indicator of the yield performance of chickpea [22].

Physicochemical and cooking properties of the two improved chickpea varieties are presented in Table 2. Arerti revealed the following values: hydration capacity of 1.07 g/g, hydration index of 2.05, swelling capacity 2.12 ml/g, and swelling index of 4.08. Similarly, Natoli variety also was characterized by hydration capacity of 1.03 g/g, hydration index 1.85, swelling capacity 1.94 ml/g, and swelling index of 3.77. The hydration capacity, swelling capacity, and swelling index were significantly (P<0.05) different between the varieties. Arerti variety had the greater hydration capacity, swelling capacity, hydration index, and swelling index than Natoli varieties. These values are close to those of Pakistani varieties [23]. Unhydrated seeds of Arerti chickpea were 1.64% and those of Natoli were 14.75% (Table 2). These values had significant (P<0.05) difference between them. Unhydrated seeds are undesirable and indicate the variation in hydration capacity between varieties as well as their respective cooking time.

Cooking time: The cooking time of the two improved chickpea varieties are shown in Table 2. The values exhibited significant (P<0.05) difference between the varieties with cooking times of 21.00 min for Arerti and 246.33 min for Natoli. The cooking time of the Arerti was very much lower than that of Natoli. Hence, Arerti variety would require less fuel energy and should be promoted to consumers. Natoli variety showed hard-to-cook (HTC) characteristic due to high number of unhydrated seeds and excessively long time it requires to cook. The cooking times observed for Arerti of Kabuli variety in this study were much less than the results reported by [24], 32.5-42.5 and 31-34 min for Desi and Kabuli varieties, respectively, of India. In another study [25]. The cooking time of the whole grains of fresh chickpeas in Netherlands varied from 112 to 142 min. The seed coat is the major barrier to chickpea softening during cooking and its contribution to cooking time exceeded that of the cotyledon in the fresh samples [26]. The cotyledon's contribution to cooking time increases with storage time [27].

### 3.2. Effect of Processing Methods on Mineral Composition of Improved Chickpea Varieties

Statistically significant (P<0.05) reduction occurred in zinc contents after the different methods of chickpea processing (Table 3). The average zinc content of raw unprocessed samples was 7.06 mg/kg whereas that of the dehulled one was 4.48 mg/kg. Boiling also resulted in lower zinc content. This reduction could be attributed to the leached minerals from the chickpea seeds into the water at different rates during water related treatments. In the dehulled samples, the average zinc content of chickpea seed was reduced due to removal of seed coat. This reduction of zinc due to dehulling was in agreement with the finding of chickpea varieties of Saudi Arabia [28]. Processing methods had significant (P<0.05) effect on iron content. The average iron content of the raw chickpeas (10.68 mg/kg) was significantly higher than that obtained after processing by different processing methods (Table 3). Soaking with the value of 8.90 mg/kg and boiling of the value of 9.03 mg/kg had no difference between them but were significantly higher than the value obtained by dehulling (8.52 mg/kg). Similar reduction of iron content occurred in Indian chickpeas with the processing methods of germinating, boiling, pressure cooking, and roasting [29]. Similar results were also observed in Egypt mung bean seeds with the processing methods of dehulling, soaking, germination, boiling, autoclaving, and microwave cooking [30].

The average calcium content of the raw chickpeas, 1267.33 mg/kg, was statistically (P<0.05) the highest as compared to those obtained by different processing methods (Table 4). The maximum reduction of calcium content occurred in dehulling process (536.56 mg/kg). This might be due to the loss of the hull as minerals are more concentrated in the testa rather than in the cotyledon [31]. Processing methods showed similar effects on calcium contents in mung bean seeds [30]. The results of this study were higher than those reported in Egyptian chickpea varieties with values of 166, 131, and 124 mg/kg after germinating, microwave cooking, and boiling, respectively [32].

### 3.3. Effect of Processing Methods on Antinutritional Factors of Improved Chickpea Varieties

Processing methods exhibited significant (P<0.05) effect on tannin content of chickpea (Table 4). The tannin content was reduced by 25.00, 50.00, 68.75, 75.00, and 82.25% due to dry roasting, soaking, dehulling, germinating, and boiling, respectively. As indicated in Table 4, boiling processing method had the highest reduction tannin in chickpeas. Tannin is a water-soluble phenol compound and predominantly found in seed coat. Thus, tannin leached out from chickpea, moreover, increasing tannin solubility. Significant tannin reduction in chickpea varieties by processing methods in this study was comparable to tannin reduction in improved dry beans studied by [16] and Indian chickpea varieties reported by [29].

Processing reduced the phytic acid (PA) content significantly (P<0.05) (Table 4). The phytic acid content was reduced by 5.89, 15.20, 31.00, 36.42, and 57.35% as a result of dry roasting, soaking, germinating, dehulling, and boiling, respectively. The maximum reductions of phytic acid, which occurred due to boiling process, were due to leaching out into soaking water [1]. In addition, germinating also reduced the phytic acid content in chickpea flour due to increase in the activity of endogenous phytase for its use as source of inorganic phosphate during germination [33].

## 4. Conclusions

According to the finding of this study, Arerti of Kabuli chickpea variety had shown low antinutritional factors and better physicochemical composition properties such as low cooking time than Natoli of Desi variety. All processing methods were effective in reduction of antinutritional factors; however, boiling was found to be best for reduction of antinutritional factors. This indicated that improved Arerti of Kabuli chickpea flour if applied in the food processing industries is commendable in production of quality food formulation especially conventional flours which are low in protein in order to increase utilization of improved chickpea flour, thereby alleviating protein malnutrition.

## Figures and Tables

**Table 1:** General characteristics of the seeds of the two chickpea varieties.

Varieties	Type	Pedigree	1000 Seed weight (g)	Seed density (g/ml)
Arerti	Food	X87TH186	260.69 ±1.10^b^	3.48 ± 0.12^a^
		/TCC14198		
		XFLIP 82-150c		
Natoli	Food	ICCX-910112-6	280.65 ± 2.38^a^	3.61 ± 0.24^a^

CV			1.19	9.25
LSD			7.29	0.74

CV= coefficient of variation; values are mean ± SE and mean values followed by the same letter in column are not significantly different at 5% level of significance; LSD = least significance difference.

**Table 2:** Physicochemical and cooking properties of improved chickpea seed varieties.

Varieties	Hydration Capacity g/g	Swelling Capacity ml/g	Hydration Index	Swelling index	Unhydrated Seed (%)	Cooking Time (min)
Arerti	1.07 ± 0.00^a^	2.12 ± 0.03^a^	2.05 ± 0.05^a^	4.08± 0 .09^a^	1.64 ± 0.05^b^	21.00 ± 2.52^b^
Natoli	1.03 ± 0.01^b^	1.94 ± 0.02^b^	1.85 ± 0.06^a^	3.77± 0.02^a^	14.75 ± 0.53^a^	246.33 ± 2.33^a^

CV	0.78	1.33	4.72	3.02	7.97	3.15
LSD	0.02	0.06	0.21	0.27	1.48	9.53

LSD = Least significance difference, CV= coefficient of variation; values are mean ± SE and mean values followed by the same letter in column are not significantly different at 5% level of significance.

**Table 3:** Effects of processing methods on mineral composition of improved chickpea varieties.

Processing Methods	Zinc (mg/kg, db)	Iron (mg/kg, db)	Calcium (mg/kg, db)
Raw (control)	7.06 ± 0.05^a^	10.68 ± 0.10^a^	1267.33± 124.47^a^
Dry roasting	5.47 ± 0.08^d^	10.17 ± 0.03^b^	844.83 ±19.82^e^
Dehulling	4.48 ± 0.08^e^	8.52 ± 0.08^e^	536.56 ± 52.56^f^
Soaking	5.64 ± 0.31^c^	8.90 ± 0.03^d^	899.00 ± 10.80^d^
Germination	5.85 ± 0.14^b^	9.39 ± 0.03^c^	930.24 ± 27.83^c^
Boiling	5.48 ± 0.03^d^	9.03 ± 0.06^d^	1035.68 ± 57.97^b^

CV	1.14	1.26	0.76
LSD	0.11	0.20	11.78

CV= coefficient of variation; values are mean ± SE and mean values followed by the same letter are not significantly different at 5% level of significance; LSD = least significance difference.

**Table 4:** Effect of processing methods on the antinutritional factors.

Processing Methods	Tannin (%)	Reduction (%)	Phytic acid ( mg/100g)	Reduction ( %)
Raw (control)	0.16 ± 0.01^a^	-	88.28 ± 1.74^a^	-
Dry roasting	0.12 ± 0.01^b^	25.00	83.08 ± 0.77^b^	5.89
Dehulling	0.05 ± 0.01^d^	68.75	56.13 ± 2.11^e^	36.42
Soaking	0.08 ± 0.01^c^	50.00	74.86 ± 1.31^c^	15.20
Germinating	0.04 ± 0.01^d^	75.00	60.91 ± 1.79^d^	31.00
Boiling	0.03 ± 0.00^e^	82.25	37.65 ± 1.81^f^	57.35

CV	14.84		4.07	
LSD	0.02		4.58	

CV= coefficient of variation; values are mean ± SE and mean values followed by the same letter in column are not significantly different at 5% level of significance; LSD = least significance difference.

## Data Availability

The data used to support the findings of this study are available from the corresponding author upon request.
